# Effect of Hydrotalcite on Indometacin-Induced Gastric Injury in Rats

**DOI:** 10.1155/2019/4605748

**Published:** 2019-04-11

**Authors:** Yan Fei Fang, Wen Li Xu, Lan Wang, Qing Wu Lian, Li Feng Qiu, Hui Zhou, Shu Jie Chen

**Affiliations:** ^1^Sir Run Run Shaw Hospital, Zhejiang University School of Medicine, Department of Gastroenterology, Hangzhou, Zhejiang 310016, China; ^2^Institute of Gastroenterology, Zhejiang University, Hangzhou, Zhejiang 310016, China; ^3^Department of Infectious Diseases, The Second Affiliated Hospital, Zhejiang University School of Medicine, Hangzhou, Zhejiang 310009, China

## Abstract

**Background and Aims:**

Hydrotalcite plays an important role in the therapy of gastric ulcer induced by nonsteroidal anti-inflammatory drugs (NSAIDs), but little is known about the mechanism. We designed two experiments to study the preventive and curative effects of hydrotalcite on NSAIDs-related gastric injury in rats and to investigate the relationship between the protective and curative mechanism of hydrotalcite and the secretion of epidermal growth factor (EGF)/prostaglandin E2 (PGE2).

**Methods:**

Two experiments were separately designed to evaluate the preventive and curative effects of hydrotalcite. A total of 25 male rats and 25 female rats were randomly divided into five groups (vehicle group, model group, omeprazole group, hydrotalcite group, and ranitidine group) in each experiment. Rats were treated with indomethacin by gavage to build the model of acute gastric mucosal injury. The concentrations of EGF and PGE2 in blood specimens and mucosal injury indexes by gross inspection were measured and an immunohistochemical technique was also employed to test the levels of EGF, cyclooxygenase-1 (COX-1), and cyclooxygenase-2 (COX-2) in gastric mucosa.

**Results:**

Comparing with model group in both preventive and curative experiments, hydrotalcite decreased the gastric injury in the mucosa of stomach significantly (7±4.5 vs. 16±11.25, 1.5±2 vs. 2.5±6;* P*<0.01*, P*<0.05). The levels of EGF and PGE2 in blood serum were markedly higher in hydrotalcite group than that in model group and ranitidine group in preventive experiment (574.39±34.28 vs. 486.22±41.73, 488.07±24.44;* P*<0.01,* P*<0.01). The expression levels of COX-2 in gastric mucosa were also higher in hydrotalcite group than that in model group in both preventive and therapeutic experiments (12±4 vs. 9±6, 14±7 vs. 9±4;* P*<0.01*, P*<0.05).

**Conclusions:**

Hydrotalcite promotes gastric protection and healing via several mechanisms, including increased levels of PGE2 in blood serum, activation of EGF, and antagonising the inhibition of cyclooxygenase (COX) caused by NSAIDs.

## 1. Introduction

Nonsteroidal anti-inflammatory drugs (NSAIDs) have been widely used in clinical fields characterized by anti-inflammatory, pain-relief, antiplatelet aggregation, and antithrombogenesis [1]. In recent years, these drugs have also been applied to prevent tumors by stimulation of apoptosis and inhibition of cell proliferation [2, 3]. With the increased use of NSAIDs, gastrointestinal side effects have also increased. NSAIDs lead to gastrointestinal toxicity mainly by inhibiting cyclooxygenase (COX), which is well known as the major protective factor of gastrointestinal system. To our knowledge, gastric mucosal injuries caused by NSAIDs are mainly shown as no symptoms, dyspepsia, mucosal erosion, ulcer, and even bleeding or perforation. Among these, erosive-hemorrhagic gastritis (20%-40%) and peptic ulcer (10%-30%) are the most common diseases.

It is of importance to prevent and treat the gastric injury caused by NASIDs and many drugs have been researched now, like omeprazole, ranitidine, and hydrotalcite. However, the superior drug to prevent and treat the gastric injury induced by NSAIDs remains controversial in clinical field. As one of these recommended drugs, hydrotalcite is widely used in clinical practice. Previous studies [4] reported that hydrotalcite has protective effects on the experimental gastric mucosal injury, and it is connected with the rise of TFF2 and c-fos protein. However, the curative properties of hydrotalcite on gastric ulcers are not fully understood and more appropriate options in drug use to defend the gastric injury caused by NASIDs also need to be further explored.

The aim of this study was to investigate the antiulcerative and curative effects of hydrotalcite and possible mechanisms. Our study helps to recommend a superior preventive and therapeutic drug to NSAIDs-related gastric injury in clinical practice.

## 2. Materials and Methods

### 2.1. Animal Experiment

The protocols for animal research were systematically evaluated by ethical and scientific care committee and approved by Run-Run-Shaw hospital affiliated with medical college, Zhejiang University. One hundred Wistar rats (200-220g) with both sexes half were obtained from Shanghai Slack laboratory animal co., LTD (SCXK:2012-002). During the experiment, rats were specifically kept in a pathogen-free state, maintained in a strict light cycle (lights on at 08:00 hours and off at 20:00 hrs) at 20-26°C and 40-70% relative humidity. After 24h of food starvation, animals were treated with indometacin (30mg/kg dissolved in ddH_2_O) by gavage to establish the model of acute gastric mucosal injury as previously described.

Two experiments were applied to evaluate the preventive and therapeutic effects of hydrotalcite in rats derived from indometacin-induced gastric injury, respectively. 50 rats with both sexes half were randomly divided into 5 groups (vehicle group, model group, omeprazole group, hydrotalcite group, and ranitidine group) in each experiment.

*(a) Preventive Experiment. *The animals were initially pretreated with distilled water (vehicle group), distilled water (model group), omeprazole (omeprazole group), hydrotalcite (hydrotalcite group), and ranitidine (ranitidine group) by gavage after 24h of food starvation. 0.5 hour later, indometacin was administrated by gavage to make the model on model group, omeprazole group, hydrotalcite group, and ranitidine group. Six hours after indometacin ingestion, rats were narcotized with 4% chloral hydrate by intraperitoneal injection, followed by collection of blood and tissue samples.

*(b) Therapeutic Experiment. *The second day after modeling induced by indomethacin (except vehicle group), drugs were administrated to groups once a day for 3 days (vehicle group was treated with distilled water once a day for 4 days) by gavage. Then after 24h of food starvation, rats were narcotized with 4% chloral hydrate by intraperitoneal injection, followed by collection of blood and tissue samples.

### 2.2. Detection of Blood Specimen

To measure the concentrations of PGE2 and EGF (pg/ml) in blood serum, blood was obtained from arteria femoralis in rats and centrifuged (3000rpm) for 10min. After separation, the specimens were cryopreserved in vials at -20°C, followed by analysis of ELISA.

### 2.3. Mucosal Lesions Analysis

To analyse the megascopic gastric lesions, the stomachs were excised and spread along the greater curvature. After repeatedly rinsing with ice saline, the area of visible erosive lesions was gauged using vernier caliper. Gastric lesions were scored by a previously described scoring system [5] as follows: one point, <1mm of erosion; two points, 1-2mm of erosion; three points, 2-3mm of erosion; four points, 3-4mm of erosion; five points, >4mm of erosion. If the erosive diameter is >2mm, the score is double. Then lesion index was calculated on the totally accumulated points.

### 2.4. Histological Analysis

The stomach tissues were excised from Wistar rats and washed with ice saline. Later, the samples were fixed in 10% neutral buffered formalin, routinely processed with dehydration, and imbedded in paraffin wax. Then the tissues were sectioned into 5*μ*m thick slices, stained with hematoxylin and eosin (H&E). Finally, the levels of EGF, COX-1, and COX-2 in gastric mucosa were measured by employing an immunohistochemical technique.

### 2.5. Statistical Analysis

One-way ANOVA and Kruskal-Wallis H (or U) rank-sum test were used to identify significant differences between groups (SPSS for Windows, Release 13.0). All values are reported as the means±standard deviation (SD), and differences were considered to be significant when P< 0.05.

## 3. Results

### 3.1. Preventive Effects of Hydrotalcite on Indometacin-Induced Gastric Injury

**Figure 1:**
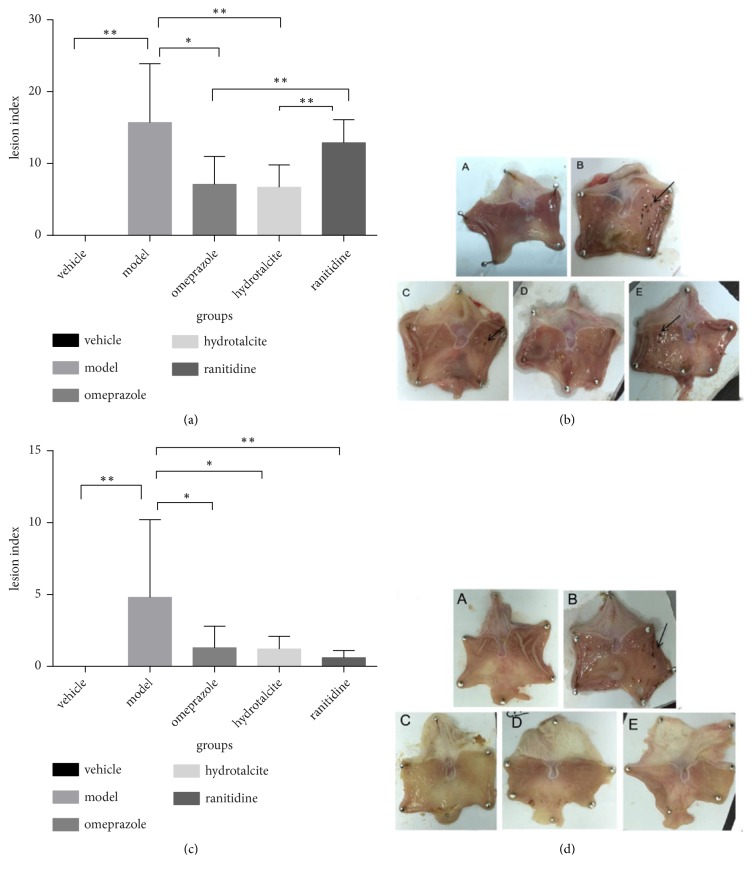
*Preventive and therapeutic effect of hydrotalcite against indomethacin-induced gastric injury in rats.* (a) Omeprazole, hydrotalcite, ranitidine, or vehicle was taken preventively by oral gavage before 30mg/kg indomethacin was given. Omeprazole and hydrotalcite significantly decreased the gastric injury in the mucosa of stomach, compared with the model group. (b) Stomach lesions in preventive experiment. A: vehicle group, the gastric mucosa was smooth, the color was ruddy, and the mucosal fold was clear. B: model group, obvious hyperemia and edema with mucosal defects that were spot hemorrhage and dispersed to all stomach surfaces. C: omeprazole group, a little spot-shaped erosion with mild hyperemia. D: hydrotalcite group, slight erosion like C. E: ranitidine group, linear ulcer lesion. Arrows indicate the area of hemorrhagic lesions in the inner surface of the stomach. (c) Gastric injury was induced by oral administration of 30mg/kg indomethacin, and then different drugs or vehicle was treated by oral gavage for 3 days. Omeprazole, hydrotalcite, and ranitidine treatment all significantly decreased the gastric injury in the mucosa of stomach, compared with the control group. (d) Stomach lesions in therapeutic experiment. A: vehicle group, the gastric mucosa was smooth, the color was ruddy, and the mucosal fold was clear. B: model group, a great deal of blood clot on the surface of mucosa, hyperemia and edema on mucosa obviously. C: omeprazole group, pale mucosa and only a small amount of dot erosion. D: hydrotalcite group, like C. E: ranitidine group, like C. Arrows indicate the area of hemorrhagic lesions in the inner surface of the stomach. Values are expressed as means±SD. *∗P*<0.05, *∗∗P*<0.01.

Macroscopic examination showed gastric lesions in the stomachs of all rats in the 5 groups in preventive experiment. Hemorrhagic lesions inside the stomach appeared in 4 groups (model group, omeprazole group, hydrotalcite group, and ranitidine group). However in vehicle group, the macroscopic examination showed clear and smooth gastric mucosa without injury. Compared with model group and ranitidine group, the lesion indexes in omeprazole and hydrotalcite groups were obviously decreased. No differences were shown in comparisons between model group and ranitidine group (Mann-Whitney U rank-sum test, Figures 1(a) and 1(b)).

### 3.2. Curative Effect of Hydrotalcite on Indomethacin-Induced Gastric Injury

To confirm the curative effect of hydrotalcite against indomethacin-induced gastric injury, gastric damage was induced by oral administration with a single dose of 30 mg/kg indomethacin, and then different drugs or vehicle was treated by oral gavage for 3 days. Gastric lesions were estimated by measuring the lesion areas on the gastric mucosal surface in all experimental groups. As shown in Figure 1(d), oral administration of indomethacin (model group) prominently aggravated the gastric injury in the mucosa of stomach, compared with the untreated normal group (vehicle group) (*∗∗*P<0.01). However, three treatment groups for 3 days markedly attenuated the gastric injury compared with the model group. Hydrotalcite, omeprazole, and ranitidine had similar therapeutic effects on healing mucosal injury (Figures 1(c) and 1(d)).

### 3.3. PGE2 and EGF Analyses in Preventive Experiment

**Figure 2:**
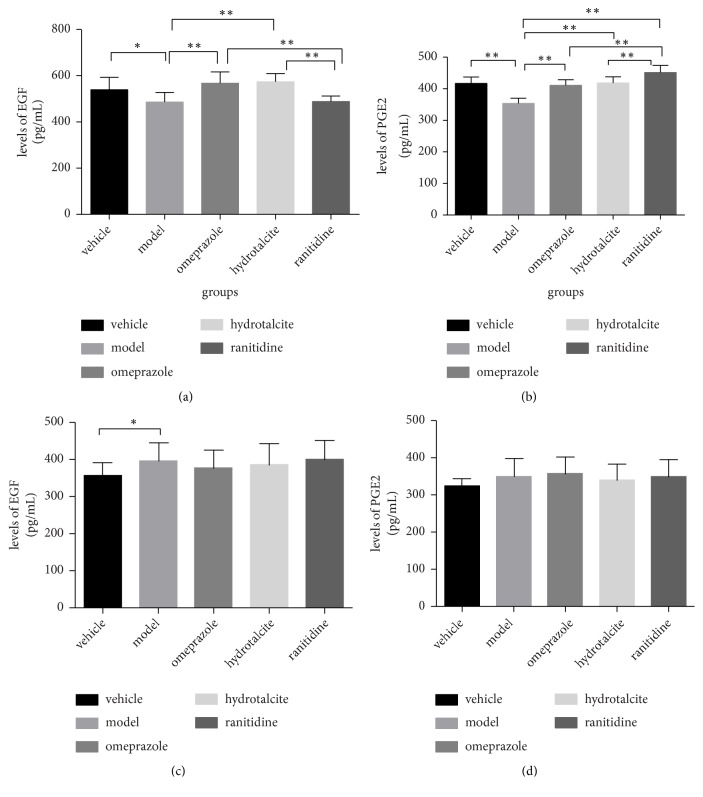
(a) Levels of EGF in blood serum in preventive experiment. (b) Levels of PGE2 in blood serum in preventive experiment. (c) Levels of EGF in blood serum in therapeutic experiment. (d) Levels of PGE2 in blood serum in therapeutic experiment. Results are the means±SD (N=10), ^*∗*^*p* < 0.05 and *∗∗p*<0.01.

PGE2 and EGF are well recognised to have anti-inflammatory effect, so we measured the concentrations of PGE2 and EGF (pg/ml) in blood serum. As seen in Figure 2(a), the concentrations of EGF were markedly reduced in model group compared with vehicle group (^*∗*^*p* < 0.05, t-test). In omeprazole and hydrotalcite groups, the concentration of EGF was higher than that in model group. However, the concentration of EGF in ranitidine group was similar to the level in model group.

As shown in Figure 2(b), the concentrations of PGE2 in omeprazole, hydrotalcite, and ranitidine groups were obviously increased when compared to the model group. The concentration of PGE2 was the highest in ranitidine group, and it was significantly higher in ranitidine group than that in omeprazole and hydrotalcite groups.

### 3.4. PGE2 and EGF Analyses in Therapeutic Experiment

In therapeutic experiment, the concentrations of EGF were significantly elevated in model group when compared to the vehicle group (^*∗*^*p* < 0.05, t-test), and comparison between other groups showed no significant difference. Although the concentrations of PGE2 in model group were higher than in vehicle group, they had no significant differences. The concentrations of PGE2 in the three drug-administration groups were similar (Figures 2(c) and 2(d))

### 3.5. Immunohistochemistry of EGF, COX-1, and COX-2 in Preventive Experiment

The gastric tissues obtained from the indometacin-induced gastric ulcer model and vehicle model were used for immunohistochemical localization. In vehicle group, model group, and other drug-taken groups, the expression levels of EGF were similar, which had no significant difference (Mann-Whitney U rank-sum test).

**Figure 3:**
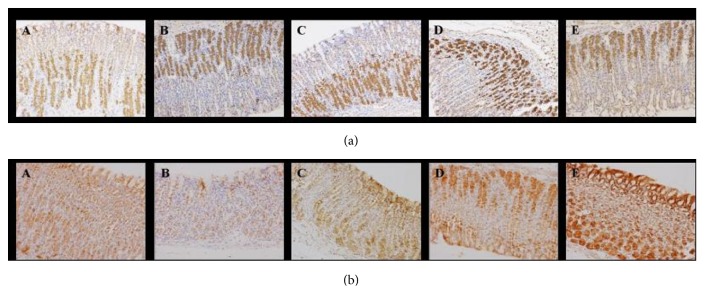
(a)* The expression levels of COX-1 were detected by immunochemistry analysis in preventive experiment.* Representative photos were shown in vehicle, model, omeprazole, hydrotalcite, and ranitidine groups. (b)* The expression levels of COX-2 were detected by immunochemistry analysis in preventive experiment.* Representative photos were shown in vehicle, model, omeprazole, hydrotalcite, and ranitidine groups.

As shown in Table 1, the expression levels of COX-1 in gastric mucosa were similar in vehicle and model groups. However, the expression levels of COX-1 in omeprazole and hydrotalcite groups were elevated in the gastric mucosa compared with ranitidine group (Figure 3(a)).

As seen in Table 2, the expression level of COX-2 in model group was obviously reduced compared with vehicle group (^*∗*^*p* < 0.05). In hydrotalcite and ranitidine groups, the expression levels were elevated significantly compared with model group (Figure 3(b)).

### 3.6. Immunohistochemistry of EGF, COX-1, and COX-2 in Therapeutic Experiment

**Figure 4:**
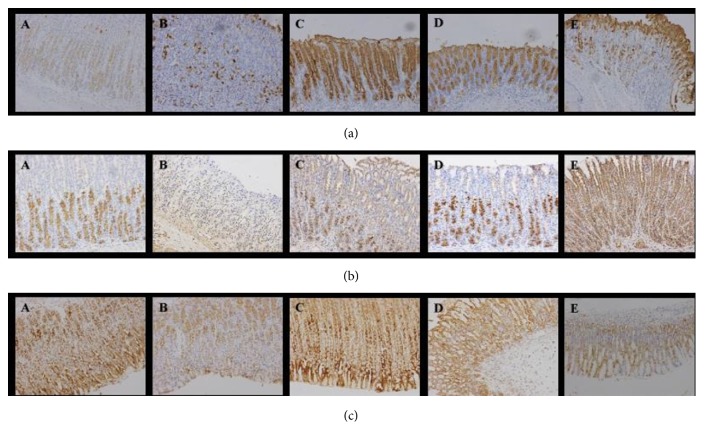
*(a) Immunohistochemical staining of EGF in gastric mucosa in therapeutic experiment (200X).* Positive cells were stained brown-yellow.* (b) Immunohistochemical staining of COX-1 in gastric mucosa in therapeutic experiment (200X).* Positive cells were stained brown-yellow. Labels are the same as Figure 4(a). The expression of COX-1 in ranitidine group was the highest.* (c) Immunohistochemical staining of COX-2 in gastric mucosa in therapeutic experiment (200X).* Positive cells were stained brown-yellow. Labels are the same as Figure 4(a). The expression of COX-2 in omeprazole group was the highest. A: vehicle group, B: model group, and C: omeprazole group, the expression of EGF was the highest. D: hydrotalcite group, E: ranitidine group.

As seen in Table 3, the expression levels of EGF in ranitidine and hydrotalcite groups were obviously lower compared with omeprazole group (Figure 4(a)). As shown in Table 4, the expression level of COX-1 in model group was the lowest, and the expression levels of COX-1 in ranitidine and hydrotalcite groups were obviously increased when compared to model group. In addition, the expression level of COX-1 in ranitidine group was the highest (Figure 4(b)).

As shown in Table 5, the expression level of COX-2 in model group was reduced significantly compared with vehicle group (^*∗∗*^*p* < 0.01). The expression levels of COX-2 in omeprazole, hydrotalcite, and vehicle groups were similar (Figure 4(c)).

## 4. Discussion

In China, about 3.6% of adults were estimated to use nonsteroidal anti-inflammatory drugs (NSAIDs) periodically [5]. NSAIDs have some common side effects, such as gastric intestinal bleeding, peptic ulcer formation, and kidney diseases [6–8]. It was previously reported that 19% of patients with NSAID use have a history of ulcer bleeding in 6 months [9]. To date, it is well known that inhibition of COX-1 by NSAIDs can partially cause gastric mucosal vulnerability while it is connected with the anti-inflammatory and analgesic effects [10–12]. Indomethacin is one of the most common drugs to make an ulcerative model by producing gastric mucosal damage [13]. Some conflicting conclusions about the ulcerogenic mechanisms of indomethacin have been reported [13]. It has been revealed that indomethacin induces gastric injury by inhibiting the release of COX-1, PGE2, and mucus; delaying of angiogenesis; increasing aggressive factors such as acid. Nevertheless, some drugs that arrest ulcer progression have been reported to heal or prevent indomethacin-induced ulcer without affecting these molecules above. Hence it was recognised that the mechanisms of NSAIDs-induced ulcer and the preventing or therapeutic effects of antiulcer drugs were still unclear. Our research was produced to understand the mechanism of hydrotalcite (one of antiulcer drugs) protecting and preventing effects on gastric mucosa.

Epithelial growth factor (EGF) was reported to play an important role in healing gastric mucosa, including cell restoration, migration, and epidermization in recent years [14, 15]. It was extensively elucidated that EGF induced the JAK/STAT signaling pathway and the phosphatidylinositol pathway, which is connected with immunity and proliferation [16]. A remarkable reduction of the EGF concentration has been reported in the gastric ulcers in a large sample of patients [17]. In our preventive experiment, the amount of EGF in hydrotalcite group was upregulated markedly compared with model group. The results showed that hydrotalcite enhanced the expression of EGF in the serum of rats evidently, which has no conflict with researches above. In this study, the expressions of EGF in 5 groups by immunohistochemistry were similar in preventive experiment, which might result from the execution of rats immediately only after 6h of indometacin-gavage. In our therapeutic experiment, it is shown that EGF takes an important role in reparative process of gastric mucosal damage in rats. It is presumed that the potential mechanism of curative effect of hydrotalcite is concerned with activating the epidermal growth factor in gastric mucosa, which is in accord with the results of one research [18].

Cyclooxygenase (COX) is the key enzyme to translate arachidonic acid to prostaglandin (PG), including 2 isomerases at least: COX-1 and COX-2. COX-1 is mainly involved in maintaining the completeness of gastric mucosa and regulation of gastric acid secretion and blood flow, while COX-2 takes part in repair process of damaged mucosa via promoting the production of PG in inflammatory response [19–23]. NSAIDs inhibit COX to reduce the synthesis of PGE2, which causes the damage of gut mucosa [24]. In this study, the positive expressions of COX-1 and COX-2 are mainly shown in parietal cells, neck cells, and epithelial cells in gastric mucosa. The expression levels of COX-1 and COX-2 in hydrotalcite group were increased obviously compared with model group in therapeutic experiment, and they were similar to the expression in vehicle group. It is presumed that hydrotalcite can resist the inhibition of COX induced by NSAIDs, which attenuated the expression of COX to normal levels and repaired the gastric mucosal damage.

As one of the main metabolites of arachidonic acid, prostaglandin E2 (PGE2) is well recognised as protective cell factor to repair damaged gastric mucosa via inhibiting the secretion of gastric acid, increasing the blood flow in gastric mucosa, and promoting the synthesis of protein and cellular renewal [25, 26]. A previous study reported that the augmentation of PGE2 stimulated antioxidative and mucin modulating properties in a rat model [27]. Our results showed that the expression of PGE2 in each drug-treated group was increased in both experiments, and the expression of PGE2 in hydrotalcite group was significantly increased compared with model group in preventive experiment, suggesting that hydrotalcite enhances PGE2 expression in the mucosal blood to protect and heal gastric mucosal injury.

Considering that the lesion index in hydrotalcite group was minimal comparing with other drug-treated groups in both experiments, the concentrations of these protective factors were not the highest in hydrotalcite group. We evaluated that there is possible involvement of other factors in the protection exerted by hydrotalcite. Previous study [28] reported that cysteamine protects gastric epithelial cell monolayers against drug induced damage, and it seems to be directly related to the activity of its sulphydryl group possibly against toxic oxygen radicals. We considered that hydrotalcite with sulphydryl compounds may exert its protective actions via its sulphydryl group by this mechanism and this hypothesis requires further research.

## 5. Conclusion

Our findings showed that hydrotalcite prevents and cures the damage of gastric mucosa in rats induced by indometacin to some extent. The potential mechanism is associated with stimulating the secretion of EGF, antagonising the inhibition of COX caused by NSAIDs, and recovery of PGE2 synthesis. Comparing with omeprazole and ranitidine, hydrotalcite is superior in protecting and healing gastric injury induced by NSAIDs.

## Figures and Tables

**Table 1:** Immunohistochemical results of COX-1 in gastric mucosa of rats in preventive experiment.

Group	N	Median of COX-1 dyeing integral	IQR
Vehicle	10	6	2
Model	10	8.5	3
Omeprazole	10	8^a^	0.5
Hydrotalcite	10	12^b^	6
Ranitidine	10	6	2

^a^ *P* < 0.05  vs. ranitidine, ^b^*P* < 0.01  vs. ranitidine.

**Table 2:** Immunohistochemical results of COX-2 in gastric mucosa of rats in preventive experiment.

Group	N	Median of COX-2 dyeing integral	IQR
Vehicle	10	12	4.5
Model	10	9^a^	6
Omeprazole	10	12	2.3
Hydrotalcite	10	12^b^	4
Ranitidine	10	16^bc^	4

^a^ *P* < 0.05  vs. vehicle, ^b^*P* < 0.01  vs. model, and ^c^*P* < 0.01  vs. omeprazole.

**Table 3:** Immunohistochemical results of EGF in gastric mucosa of rats in therapeutic experiment.

Group	N	Median of EGF dyeing integral	IQR
Vehicle	10	0	4
Model	10	6^a^	2
Omeprazole	10	9^b^	3
Hydrotalcite	10	9^cd^	3.5
Ranitidine	10	9^bd^	1

^a^ *P* < 0.01  vs. vehicle, ^b^*P* < 0.01  vs. model, ^c^*P* < 0.05  vs.model, and ^d^*P* < 0.05  vs. omeprazole.

**Table 4:** Immunohistochemical results of COX-1 in gastric mucosa of rats in therapeutic experiment.

Group	N	Median of COX-1 dyeing integral	IQR
Vehicle	10	7	3
Model	10	3^a^	4.5
Omeprazole	10	6	3
Hydrotalcite	10	7^b^	3
Ranitidine	10	8.5^cde^	4

^a^ *P* < 0.05  vs. vehicle, ^b^*P* < 0.05  vs. model, ^c^*P* < 0.01  vs. model, ^d^*P* < 0.05  vs. omeprazole, and ^e^*P* < 0.05  vs. hydrotalcite.

**Table 5:** Immunohistochemical results of COX-2 in gastric mucosa of rats in therapeutic experiment.

Group	N	Median of COX-2 dyeing integral	IQR
Vehicle	10	14	4
Model	10	9^a^	4
Omeprazole	10	16^b^	4
Hydrotalcite	10	14^c^	7
Ranitidine	10	8.5	8.5

^a^ *P* < 0.01  vs. vehicle, ^b^*P* < 0.01  vs. model, and ^c^*P* < 0.05  vs.model.

## Data Availability

The data used to support the findings of this study are available from the corresponding author upon request.
